# Crossing to Safety

**DOI:** 10.4269/ajtmh.19-0159

**Published:** 2019-07

**Authors:** Claire Panosian Dunavan

**Affiliations:** Division of Infectious Diseases, Department of Medicine, David Geffen School of Medicine at UCLA, Los Angeles, California

When Didi (not her real name) first came to the clinic, the fiery nodules on her face barely hinted at the strange array of lesions still covered by her clothes. Succulent and irregular, a rash on her knee resembled a dying summer rose. Another on her thigh was a ragged, tremulous ring.

After completing our examination, several of us shook Didi’s hand. You’ll get better, we comforted the small, earnest woman. And see? Touching you is fine. Try not to worry. You will come here often, and we are always available by phone.

Back in the hall, we foamed our hands and talked. “Her disease is unstable,” our lead doctor sighed. “Treatment could be rough.”

By then, Didi’s diagnosis was 90% certain. Two weeks earlier, an outside dermatologist had biopsied Didi’s skin and ordered acid-fast stains, then called our publicly funded clinic. After scanning our new patient’s slides through a high-powered lens, we had no further doubt. Their curved, red bacilli spelled the ancient scourge of leprosy, also called Hansen’s disease.

Our next task was to classify Didi’s infection. Did its clinical and microscopic clues match the crusty patches and sparse bacteria of tuberculoid leprosy? No, they did not. Nor did they fit lepromatous disease, the infection’s opposite pole. Lepromatous patients are immunologically numb to their invaders. As a result, along with their many thickened blotches often paired with scalloped pinnae, missing eyebrows, and swaths of anesthetic skin, their initial biopsies contain wall-to-wall clumps of bacilli as far as the eye can see.

Didi’s café-au-lait skin was satin-smooth, except where her islands of woe had recently bloomed. Her eyebrows were full, her ears unscathed, her sensation intact. Most importantly, her tissue was only modestly dappled with bacilli. She had borderline leprosy, a “middle” form which is notoriously labile.

Over the next few weeks, we got to know Didi, an unaffected woman from a far-away place whose smiles often cheered us. At the same time, her private life was complex. Several years earlier, she had arrived in the United States as a hopeful, young bride; now, having left her spouse, she was living with friends and launching her life anew. “My parents didn’t want me to marry him,” she finally confided one morning. “They said he was too old.”

“What will you do next?” I asked. “Return to your family?”

“No,” she said. “For now, I am studying. Once I am better, I’ll find work and stay.”

And, for a while, Didi did get better. After faithfully downing pills for 2 or 3 months, her nodules flattened and her pains disappeared. But she also suffered side effects. So when dapsone triggered anemia and rifampin roiled her liver, we reduced her doses and added clofazimine, which turned her complexion mahogany brown. Through it all, she never complained.

Then came the holidays and a city-wide siege of flu. The next time we saw Didi, she was coughing and gaunt. And her skin! Not only had her earlier rashes flared and new ones erupted, back were her electrical pains. Near the bridge of her nose, we could even palpate a tender twig of her trigeminal nerve. Desperate to avoid a sudden ocular palsy, foot drop, or other possibly permanent injury, we added hefty doses of prednisone to suppress Didi’s “reversal reaction,” a well-known complication of borderline leprosy.

But the following week, Didi was still unwell, and she was also not alone. “Her companion doesn’t have an appointment,” our front-desk clerk whispered on the phone, “but he insists on being seen. What shall I tell him?”

“If Didi agrees, he can join her in the room,” our chief doctor said. “If he wants to ask questions, we will answer them in her presence.”

Soon, seven clinic members—medical specialists, trainees, and an occupational therapist—crowded the small, unadorned space. Did our *force majeure* intimidate Didi’s ex? On the contrary, the steel-haired man seemed to relish our attention. At the same time, he peppered us with questions (“How contagious is she?” “Will I get her disease?” “What are its earliest symptoms?”), however, he neither looked at Didi nor mentioned her health, not even to ask: “How is she doing?” or “Will she be okay?” She, in turn, sat as mute as a stone.

Hansen’s disease, named for the 19th-century Norwegian doctor who first identified its causative microbe in local patients’ tissues, is a minimally contagious infection whose incubation ranges from a few years to several decades. And this is just one of its enigmas. Today, in leprosy’s most entrenched, residual haunts (for example, in certain parts of India or Brazil), many residents have positive serum antibodies to *Mycobacterium leprae*, yet never progress to overt disease. Nor do economics fully predict outcome, especially in people who were silently infected in one country, then moved to another. Although modern leprosy largely afflicts the tropical poor, in the United States, between 100 and 200 cases are newly diagnosed every year in people from all walks of life and corners of the globe. In short, medical science has not yet identified the various biologic and genetic factors that allow one exposed person to remain well for a lifetime and another to develop life-changing illness, much less to gauge more elusive cofactors.

Nonetheless, for weeks after meeting Didi’s former husband, I continued to stew over his callous behavior. Had other toxic behaviors at home somehow tipped the balance when Didi’s blight first began to stir? This theory was impossible to test. Gender inequity, on the other hand, is not. According to recent studies from endemic locales, leprosy’s famous stigma still injures women far more than men, its price measured in everything from complete ghettoization to removal of children, exclusion from work, and prohibitions against sharing household utensils.

Then I had another crazy idea. Because nearly all of our Los Angeles patients are immigrants, had some of them succumbed to leprosy, in part, because they had left all things familiar and safe? In such an inconsistent disease, how could one ignore unseen, emotional contributions to the cryptic interplay of macrophages and lymphocytes, molecules, and receptors? Even in our busy clinic, we always asked about stress when trying to parse our patients’ clinical see-saws.

In recent years, social determinants of disease have come to include income, early childhood development, education, employment, housing, gender, and race. “Social epidemiology” goes further, stating that two or more variables can, at times, act together to bend a health outcome. Someday, I fantasize, students of infectious disease will consider nontraditional variables in patients with leprosy who are profoundly isolated, work two or three jobs, or must silently confront other obstacles and challenges while gaining their first precarious footholds in a new country.

Meanwhile, there’s the human paradox of this infection historically freighted with pain. Even while battling its microbe and mitigating its damage, modern leprosy doctors witness recoveries shaped not only by drugs but by courage and faith. These are the qualities I first observed and remember clearly from a long-ago rotation in Carville, Louisiana (the former site of the U.S. Public Health Service Hospital for Hansen’s Disease). Forty years later, my colleagues and I witness the same inspiring traits in nearly every patient we see at our weekly clinic.

Didi is no exception. Eighteen months following her diagnosis, she is much improved despite the occasional whiplash of her illness. She has also remained single and resilient while studying online, acquiring new skills, and pursuing new dreams. In fact, our patient embodies everything one might hope to find in a modern global citizen and medical survivor. Finally, although her russet-brown skin still shows traces of her early, exotic lesions, Didi never fails to express thanks each time we see her.

We respond in kind, for we have shared in something almost sacred while watching her cross to safety as a woman, an immigrant, and a person with leprosy. Not that long ago, as we rarely say out loud but never forget, her journey might have led to a far different place.

